# Associations Among Taste Perception, Food Neophobia and Preferences in Type 1 Diabetes Children and Adolescents: A Cross-Sectional Study

**DOI:** 10.3390/nu11123052

**Published:** 2019-12-13

**Authors:** Chiara Mameli, Camilla Cattaneo, Luisa Lonoce, Giorgio Bedogni, Francesca Chiara Redaelli, Maddalena Macedoni, Gianvincenzo Zuccotti, Ella Pagliarini

**Affiliations:** 1 Department of Pediatrics, V. Buzzi Hospital, Università degli Studi di Milano, 20154 Milan, Italy; chiara.mameli@unimi.it (C.M.); gianvincenzo.zuccotti@unimi.it (G.Z.); 2 Department of Food, Environmental and Nutritional Sciences (DeFENS), Università degli Studi di Milano, 20133 Milan, Italy; ella.pagliarini@unimi.it; 3 Department of Pediatrics, V. Buzzi Children’s Hospital, Università degli Studi di Milano, 20154 Milan, Italy; luisa.lonoce@unimi.it (L.L.); francescachiara.redaelli@unimi.it (F.C.R.); macedoni.maddalena@asst-fbf-sacco.it (M.M.); 4 Clinical Epidemiology Unit, Liver Research Center, Basovizza, 34149 Trieste, Italy; giorgiobedogni@gmail.com

**Keywords:** diabetes mellitus, taste, type 1 diabetes, food neophobia, liking, PROP, fungiform papillae

## Abstract

Type 1 diabetes (T1D) is one of the most common systemic diseases in childhood which predisposes the patient to serious short-term and long-term complications, affecting all body systems. Taste and olfactory impairments were first described a long time ago in adult patients affected by diabetes (both type 1 and type 2 diabetes). However, studies evaluating taste perception, behavioral attitudes (e.g., food neophobia), and preferences toward foods in children and adolescents affected by T1D are globally lacking. Therefore, the purpose of this study was to assess taste sensitivity, food neophobia, and preferences among children and adolescents affected by T1D and healthy controls in a cross-sectional study. T1D patients presented a significantly lower ability in general to correctly identify taste qualities, especially bitter and sour tastes. Moreover, they were characterized by fewer fungiform papillae compared to controls, as well as a lower responsiveness to the bitter compound 6-n-propylthiouracil (PROP). There were no significant differences in food neophobia scores between the two groups, but differences were observed in the mean hedonic ratings for some product categories investigated. Diabetic patients showed a greater liking for certain type of foods generally characterized by sourness and bitterness, an observation probably linked to their impaired ability to perceive taste stimuli, e.g., sourness and bitterness. These results may help to enhance the understanding of these relationships in populations with elevated diet-related health risks.

## 1. Introduction

Type 1 diabetes (T1D) is one of the most common systemic diseases in childhood [1] and predisposes patients to serious short-term and long-term complications, affecting all body systems [2]. Up until now, some complications have been studied and understood in more depth than others. Nevertheless, it is likely that diabetes induces a variety of deficits not yet known or investigated.

Taste and olfactory impairments were first described a long time ago in adult patients affected by T1D [3,4,5,6,7], but recent studies were not able to confirm these findings [8,9]. In particular, some authors showed that T1D was associated with a reduction in gustatory function [3,5,7], with impairments mostly found with respect to the sweet taste perception in comparison to other taste modalities [10,11]. In addition, Pavlidis and colleagues [12] found impairments in the density and morphology of the fungiform papillae (FP) among patients with diabetes, which are the gustatory anatomical structures containing taste buds.

Data in the literature suggest that the inherited ability to perceive bitter thiourea compounds, such as 6-n-propylthiouracil (PROP), may be a surrogate measure of taste perception and a marker for increased health risk. Variation in taste responsiveness to PROP has been linked to various health disorders, including obesity [13] and diabetes [14]. However, related data are still scarce and controversial, probably due to differences in the methodologies used to evaluate taste perception and the involved subjects (e.g., those affected by type 1 or type 2 diabetes).

Contradictory results were also reported when the associations between taste impairment and metabolic control, disease duration, and the presence of diabetes-related complications were studied [10,11,15]. However, this field of research remains largely unexplored.

Notably, to the best of our knowledge, studies evaluating taste perception in children and adolescents affected by T1D are lacking globally. Since T1D disease occurs mostly in this younger age group, knowledge regarding their perceptions of taste may be useful to understand the vicious cycle leading glycemic control deterioration. 

Differences in the ability to smell and taste contribute to individuals’ food choices, diet, nutrition, and may impact overall health status [16,17]. Psychological barriers are another important aspect with the ability to shape food habits, due to the complex interplay between innate and rapidly acquired taste preferences [18]. In this context, food neophobia, identified as an inherent adaptive personality trait [19], was defined as the rejection of foods that are novel or unknown to the child [18]. Food neophobia may lead children to largely increase the intake of saturated fat and to reduce the consumption of vegetables, fruit, fish, and meat [20,21,22,23], thereby affecting overall food variety and dietary quality.

Since children and adolescents affected by T1D fall short of meeting dietary guidelines, which include choosing a variety of foods as part of a healthy diet to maintain good metabolic control and long-term diabetes health outcomes [24,25], this maladaptive eating behavior, as well as reduced taste sensitivity, may further complicate adherence to overall diabetes management.

In light of the above, the aim of the present study was to compare taste perception in children and adolescents affected by T1D with healthy controls in a cross-sectional study. As additional measurements of taste sensitivity, FP density and PROP responsiveness were also assessed. We hypothesize that T1D children and adolescents are less sensitive to basic tastes and PROP compound and present lower FP density in respect to the control group. Moreover, food neophobia and preferences for specific food categories were evaluated in both groups involved in the study in order to understand whether the diabetic state could be related to a different attitude toward foods.

## 2. Material and Methods

### 2.1. Subjects

This study was performed between 7 January 2018 and 31 June 2019 with 31 consecutive patients affected by T1D, who were followed at the Diabetes Clinic of the Vittore Buzzi Children’s Hospital (Milan, Italy). The patients were regularly followed at the clinic since their diagnosis of T1D. Data used in the present study were collected during an annual visit for the screening of diabetes-related complications.

The inclusion criteria included a diagnosis of T1D, intensive insulin therapy, between 6 and 15 years of age, Caucasian ethnicity, and diabetes duration of at least 12 months. The exclusion criteria were type 2 diabetes, maturity-onset diabetes of the young, syndromic diabetes, and smoking. Moreover, we excluded patients with acute or chronic disease interfering with smell or taste function and patients taking drugs known to affect smell or taste.

Thirty-one healthy sex- and age-matched (6–15 years old) children and adolescents were recruited as the control group from other departments of the clinic while applying the same exclusion criteria. Both of the diabetic and control groups were evaluated for taste sensitivity, food neophobia, food preference, PROP responsiveness, and FP density as described below. Moreover, each T1D patient underwent a medical and neurological examination, which is described below, and a fundus oculi assessment.

The study was conducted according to the Declaration of Helsinki and was approved by the Ethical Committee of ASST-Fatebenefratelli-Sacco. Written informed consent was obtained from the parents of the children and adolescents involved.

### 2.2. Clinical Assessment

The following data were collected from each patient at the time of the study: Age, gender, ethnicity, height, weight, body mass index (BMI), age at diagnosis of T1D, disease duration, and type of insulin therapy (multiple daily injections (MDI) or continuous subcutaneous insulin infusion (CSII).

The standard deviation scores (SDS) of weight, height, and BMI were calculated using both World Health Organization (WHO) [26] and Italian reference data [27]. The pubertal stage was assessed according to the Tanner stages [28]. Tanner stages 1 and 2 were combined into a prepubertal/early pubertal category, stages 3 and 4 were defined as pubertal, and stage 5 was considered to be post-pubertal development. Systolic and diastolic blood pressures were measured following international guidelines [29]. Blood samples were obtained after an overnight fast for 10 hours. Lipid profiles, including total cholesterol (TC), high-density lipoprotein cholesterol (HDL), low-density lipoprotein cholesterol (LDL), and triglycerides (TG), were measured using standard laboratory methods. Glycated hemoglobin (HbA1c) was measured using a fully automated high-performance liquid chromatography system (Variant II, Bio-Rad Laboratories, Munich, Germany). A first morning urine sample was obtained from all patients to evaluate microalbuminuria. All samples were analyzed by the laboratory of ASST-Fatebenefratelli-Sacco.

We also performed capillary blood glucose tests on all patients before starting the taste sensitivity assessment. Patients with values between 70 and 140 mg/dL were permitted to start the tests described below. Data obtained from the medical charts included HbA1c at diagnosis and every 3 months until the day of the study, and the presence of any diabetes-related complications.

### 2.3. Taste Sensitivity Assessment

#### 2.3.1. Taste Recognition Ability

Subjects’ taste recognition ability was tested using the “taste strips” method [30]. The measurement protocol, which is fully described elsewhere [31], was successfully applied in previous studies involving young subjects [32,33], and especially in several studies and clinical contexts [8,9,34,35]. In brief, the prefabricated taste strips are 16 filter papers impregnated with sweet, salty, sour, and bitter taste solutions in four ascending concentrations, plus 2 blank strips. The taste strips were presented in a randomized order, starting with the lowest concentration. Each subject was asked to place each strip on the anterior part of the tongue, to suck it for a maximum of 20 s, and then to identify the taste quality by selecting one of five possible answers (sweet, sour, salty, bitter, no taste) on a form. Before starting and between each taste strip, the mouth was rinsed with water to minimize adaptation and to control for carry-over effect.

#### 2.3.2. PROP Responsiveness

PROP responsiveness was evaluated following the procedure described by Bartoshuk and colleagues [36]. PROP papers were prepared by soaking circular shaped pieces of filter paper that were 3 cm in diameter (Whatman #1) in a saturated solution of PROP to near-boiling temperature. Papers were air-dried and stored in small glassine envelopes. Subjects were presented with the PROP paper and were instructed to place the filter on the anterior part of the tongue, to suck it for a maximum of 20 s, and then to rate the bitterness intensity perceived using the Labeled Magnitude Scale (LMS). This scale was a semi-logarithmic 100 mm scale ranging from “barely detectable” to “strongest imaginable.” Instructions for using the scale were given according to Green and collaborators [37]. Given the young age of subjects involved, the anchor “strongest imaginable” was defined as the most intense oral sensation a child/adolescent had experienced in everyday life [38].

#### 2.3.3. FP Density

The procedure used to asses FP density was fully described elsewhere [33]. In brief, tongue pictures were collected with blue staining, coloring the interested area with a cotton-tipped applicator impregnated with a blue food colorant (F.lli Rebecchi, Color Dolci, Rivergaro, Piacenza, Italy). On the left side of the tongue, a filter paper circle (6 mm diameter) was placed approximately 1–2 cm from the tip and used as a template. Digital pictures were taken using a 16-megapixel digital camera (NIKON Corporation, Japan) in macro mode with no flash, and the best photo was selected. Using Adobe Photoshop software (Adobe Systems Incorporated, San Jose, California), three circles (of the same diameter as the filter paper template) were drawn on the anterior part of the tongue. The FP were counted inside each marked circle by two independent operators, according to Proserpio and colleagues [39]. The Denver Papillae Protocol [40] was followed to classify FP according to shape, color, size, and recession. The counts were submitted to one-way fixed ANOVA and were considered valid if the operator’s effect was not significant (*p* > 0.05). Then, the mean of the two counts was calculated.

### 2.4. Food Neophobia Assessment

The Italian Children Food Neophobia Scale (ICFNS) [41] was used to evaluate food neophobia in children and adolescents. The food neophobia scale was specifically designed for children and showed higher reliability across different countries [42]. This tool consisted of 8 items (4 related to neophobic and 4 related to neophilic attitudes) and responses were based on a 5-point scale with facial expressions (emoticons) representing different degrees of agreement (from left to right, “very false for me” (1)—a frown face with both thumbs down; “false for me”—a frown face with one thumb down; “so-so”—a neutral face with no thumbs shown; “true for me”—a smiley face with one thumb up; “very true for me” (5)—a smiley face with both thumbs up). Thus, the food neophobia score ranged from 8 to 40 (neophilic item scores were reversed) with higher scores denoting a greater level of food neophobia.

### 2.5. Food Preferences Assessment

Food preferences were measured using a 44-item food questionnaire based on a previous questionnaire proposed by Smith and colleagues [43]. Children and adolescents were asked to indicate their liking on a 7-point facial hedonic scale ranging from super bad (1) to super good (7) [44]. Each single item belonged to one of the following general categories: Vegetables (e.g., carrots, broccoli, and tomatoes), fruits (e.g., banana, peach, and apple), starches (e.g., pasta, bread, and rice), fats and oils (e.g., butter and olive oil), meat and fish (e.g., white meat, red meat, and fish), dairy products (e.g., milk and cheese), and sweet and salty snacks (e.g., chocolate, chips, and ice-cream).

### 2.6. Statistical Analysis

Most continuous variables were not Gaussian-distributed and were all reported as the 50th percentile (median) and 25th and 75th percentiles (interquartile range (IQR)). Discrete variables were reported as the number and proportion of subjects with the characteristic of interest.

To answer the main study question, i.e., whether there was any difference in gustatory function between children with and without T1D, all correctly identified taste strips of the qualities sweet, sour, salty, and bitter were summarized using a Total Taste Score (TTS), giving a maximum score of 16 points. Differences between children with and without T1D were evaluated by means of a linear regression model (LRM) with robust confidence intervals, using TTS as the outcome and T1D status (0 = no; 1 = yes) as the predictor [33]. The LRM was also used to evaluate whether there were differences in PROP responsiveness, neophobia, and food preferences between the two groups. The secondary study question, i.e., whether there was a difference in the density of FP between children with and without T1D, was tested by means of a Poisson regression model (PRM) using the density of fungiform papillae as the outcome and T1D status (0 = no; 1 = yes) as the predictor [33]. Statistical analysis was performed using Stata 16.0 (Stata Corporation, College Station, TX, USA).

## 3. Results

Thirty-three consecutive patients were eligible for the study, but two refused to participate. Therefore, from 7 January 2018 to 31 June 2019, we recruited 31 children and adolescents (male *n* = 14, 45%) and 31 healthy controls matched for sex (male: *n* = 16, 52%) and age, who fulfilled the inclusion and exclusion criteria.

The median (IQR) age was 11 (9–14) years old for patients and 10 (9–11) years old for control subjects. Among the patients, 15 (48%) were treated with CSII while *n* = 16 (52%) were treated with MDI therapy. The median (IQR) HbA1c was 8% (64 mmol/mol) (7–8%; 53–64 mmol/mol) and the median (IQR) duration of disease was 4.0 (2–7) years. There were no differences between the median (95% Confidence Interval, CI) or SDS of BMI in T1D (0.13, 95% CI: −0.30–0.57) and control subjects (0.29, −0.71–0.13, *p* = 0.181, median regression).

No patient had arterial hypertension and all patients had normal neurological evaluations. No patient had an albuminuria of >30 mg/g of creatinine/day. The median HbA1c from onset to the time of the taste evaluation was 8% (58 mmol/mol) with an IQR of 7.0–8.0% (53–64 mmol/mol). Fundus oculi evaluations were normal in all patients (see Supplementary Table S1 for in-depth characterization of T1D patients).

### 3.1. Taste Sensitivity

To measure the subjects’ taste recognition abilities, a Total Taste Score (TTS) was calculated by adding together the correct answers given by a subject when identifying all four of the taste qualities presented. The maximum score achievable by the subjects was 16. In general, patients with T1D showed significantly greater difficulty in correctly identifying the different taste qualities compared with the control subjects, resulting in a lower TTS. Table 1 reports the mean (95% robust CI) differences in the TTS and the sour and bitter taste scores between T1D and control subjects. No significant differences were found regarding the sweet and salty taste scores. The components of the main outcome (TTS) were reported for descriptive purposes only (see Supplementary Table S2 for in-depth characterization of T1D patients and control subjects).

The mean (robust 95% CI) difference in the density of FP between T1D patients and control subjects was −10 (95% CI: −12 to −8, *p* < 0.001, PRM) papillae/cm^2^, which corresponded to means (robust 95% CI) of 13.9 (12.6 to 15.2) and 23.7 (22.0 to 25.5) papillae/cm^2^. Moreover, the mean (robust 95% CI) difference in PROP responsiveness between T1D and control subjects was −17.1 (35.3 to −1.0, *p* = 0.064, LRM), which corresponded to means (95% CI) of 27.7 (16.3 to 39.0) and 44.8 (30.7 to 58.9).

The relationship between TTS and FP density was investigated. The spread of TTS was the same along all of the values of FP in both T1D and control subjects, showing a lack of association between the two variables (the wider spread among the T1D patients was uncertain due to the lack of association between TTS length and FP density). The relationship between PROP scores and FP density was also evaluated. The spread of PROP scores was the same along all of the values of FP in both T1D and control subjects, showing a lack of association between the two variables.

### 3.2. Food Neophobia and Food Preferences

No significant difference was found between the two groups regarding the food neophobia score. Nevertheless, differences were observed between the T1D patients and the control group regarding the mean hedonic ratings for some of the product categories investigated (Table 2). With the obvious limitation of the multiple exploratory tests performed among the components of the main outcome, it was of some interest that children and adolescents differed in their preference for fruits and vegetables, with patients affected by T1D giving higher preference scores than the healthy controls for these product categories. (see Supplementary Table S2 for in-depth characterization of T1D patients and control subjects).

No significant associations were found between disease duration, HbA1c measures from disease onset to the time at which taste was assessed, and TTS, PROP, and FP density.

## 4. Discussion

Consistent with the stated hypothesis (i.e., that diabetic patients would be less sensitive than the control group), the findings presented in Table 1 showed that children and adolescents with and without T1D presented differences in their taste sensitivity measures. Indeed, T1D patients showed a lower ability in general to identify taste qualities compared to the control subjects. Our results were in line with both older and more recent studies [7,10,45]. Taste impairment was found in the T1D patients relative to the control group with respect to electro and chemical gustometry [7], as well as increased thresholds for basic tastes when perception was evaluated using serially diluted solutions [10,45]. On the contrary, Naka and colleagues [9] as well as Altundag and collaborators [3], who used the same “taste strips” method applied in the present study, did not find any significant difference in taste performance between adult patients affected by uncomplicated type 1 or type 2 diabetes and healthy controls. However, it has to be mentioned that this was the first study involving children and adolescents (affected by T1D), for whom sensory performance cannot be compared with older subjects, as taste can change over the life span. The T1D subjects in our study showed significantly lower abilities to recognize both sour and bitter tastes, while no differences were highlighted for salty and sweet tastes. Data relative to the perception of basic tastes in T1M are scarce and contradictory. A higher threshold for salty taste was reported by some [45,46,47], but not by others [7]. An altered bitter taste perception was reported in older studies [7,45], but has not been confirmed since [46,47]. Similarly, an impaired sour perception was shown by some authors [45,46], but not all authors [7]. These highly contradictory results might be due to the great heterogeneity among studies regarding the enrollment of patients characterized by differences in glycemic control, to the presence of chronic complications and other possible confounders.

The underlying mechanisms of taste dysfunction in diabetes is still unknown, but some pathophysiological explanations have been proposed. Neuropathic complications, such as peripheral neuropathy with lingual nerve involvement, could be a cause of gustatory alterations in diabetic patients [7,46,48]. However, this underlying mechanism is still being debated, since taste impairments were also found in our group of T1D patients without neuropathic complications, which was in line with previous studies [49,50]. Furthermore, oral mucosal disorders, such as xerostomia and salivary flow reduction, were reported in diabetic patients [51,52,53]. Since saliva acts as a solvent for taste stimuli and transports taste molecules to taste receptors, increased dryness of the mucosa or saliva density can hamper this transport to taste buds [54]. Moreover, less salivary flow induces a reduction in gustin/carbonic anhydrase VI secretion, which acts as salivary growth factor involved in the maturation of taste papillae and the homeostasis and integrity of taste receptors [55]. This protein was also linked to perception of the bitter compound PROP [56,57]. The present study found that children and adolescents with T1D presented a reduced responsiveness to PROP and a significantly lower FP density, which was in accordance with previous results [12,58]. However, our study failed to establish a direct correlation between FP density and TTS or PROP responsiveness. The role of FP density in determining PROP bitterness intensity as well as the perception of basic taste has been extensively studied, but still remains unclear [59]. Several recent studies failed to find significant relationships [60,61].

Many studies focused on taste perception and preferences because of their direct impact on diets [62,63]. Indeed, taste impairments might lead subjects to search for foods containing more flavors, thus explaining, at least in part, the poor compliance to dietary recommendations observed in many diabetic patients [25].

The hypothesis that diabetic patients have a greater liking for certain types of taste stimuli (e.g., sweet and salty), which contributes to glycemic control deterioration, is probably linked to the different abilities to perceive taste stimuli, which was explored previously [11,50]. Indeed, an association between reduced sweet and salty taste sensitivity and greater preferences and intake for sweet and salty foods was previously reported [11,50]. However, our study failed to find this association. T1D children and adolescents seemed to prefer fruits and vegetables more than the control group. As previously stated, this difference could be due to a lower sensitivity to bitter and sour stimuli, which was exhibited by our diabetic patients. The preferences or rejection of specific food categories, such as fruits and vegetables, could be in part due to reduced or increased perception of strong and generally “warning” taste stimuli, which often characterize plant food. Most fruits and vegetables are rich in phenolic compounds, among others, which convey bitterness, astringency, and sourness in food [64]. Moreover, the children and adolescents involved in this study regularly and attentively received medical nutrition therapy (e.g., increasing the consumption of fruit and vegetables) as part of their disease management since the onset of T1D, to maintain good metabolic control and more positive long-term diabetes health outcomes. This may have led our participants to be more likely to prefer and consume these food categories to achieve greater adherence to diet-related diabetes management tasks. Experimental studies in children have shown that guidance modeling of healthy eating and regular and repeated exposures to unfamiliar food (e.g., fruits and vegetables) increases both acceptance and consumption of those exposed foods [65,66], thereby improving dietary variety.

In the present study, we also tested the hypothesis that diabetic patients were characterized by different attitudes toward food (e.g., greater neophobia). However, our results did not highlight any difference in food neophobia levels between diabetic patients and the healthy control group. To the best of our knowledge, few researchers have investigated this topic in a cross-sectional study involving subjects with T1D and healthy controls. Indeed, only Quick and colleagues [25] examined the relationship between food neophobia, pickiness, and dietary variety and quality, and diabetes management adherence and glycemic control in a consistent group of young subjects affected by T1D. The authors reported that diabetes management adherence was negatively associated with both neophobia and pickiness and positively associated with dietary variety. However, their study did not recruit a control group matched for age and gender or measure food neophobia with the same questionnaire, which was filled in by parents of the subjects and not self-administrated. Thus, it was not possible to make any kind of comparison with the present research.

We also examined the relationships between taste sensitivity and long-term metabolic control markers (median HbA1c value since the disease onset) or disease duration. We did not find any significant associations between disease duration, HbA1c, and taste perception variables, which was consistent with previous results [8,9,46,67]. Some hypotheses could be proposed to explain these results. The “diabetes” per se, independently from the duration, may induce taste impairment from onset. Chronic inflammation and cytokine alteration (excessive chronic inflammatory cytokine production) described in T1D could be pathogenesis factors involved in the development of taste alteration.

Another possible explanation for the lack of correlation between mean HbA1c and taste parameters is the role of HbA1c as a predictor of diabetes-related complications. In fact, an increasing number of studies recently showed that HbA1c has many limitations as a marker of disease control [68]. A panel of experts [69] suggested that we should try to look beyond HbA1c and search for new metrics in clinical practice to estimate the risk of diabetes-related chronic complications. In this field, the role of the “time in range” (TIR) parameter is emerging [70]. Unfortunately, we could not calculate the TIR in our study because nearly half of patients involved did not regularly use the continuous glucose monitoring system.

Finally, we cannot completely exclude the possibility that differences in cognitive ability between diabetic and healthy groups may have partially contributed to the taste recognition findings, since diabetes is known to impair cognition to some extent [71,72]. However, this interpretation should be taken with caution because, at present, evidence regarding the pediatric population is insufficient to support that youth with T1D display cognitive deficits or differences compared to control subjects.

Our findings should be interpreted in light of the study limitations. A causal interpretation of the observed associations should not be performed due to the cross-sectional design used. Moreover, findings may not be generalizable to all children and adolescents affected by T1D, since the study population was a convenient sample recruited in only one pediatric diabetes center. Finally, we did not obtain data regarding lipid profiles in the control group because the healthy population did not give the consent to the blood tests. Despite these limitations, this is the first study to examine taste sensitivity, food neophobia, and preferences among children and adolescents affected with T1D, and thus enhances our understanding of these relationships in a population with elevated diet-related health risks.

In conclusion, our study showed that children and adolescents affected by T1D had a lower ability in general to correctly identify taste qualities compared to the healthy subjects, especially with respect to bitter and sour tastes. Moreover, our patients showed a lower responsiveness to the PROP compound and a reduction in FP density that was independent of disease duration and metabolic control. Moreover, diabetic patients showed a greater liking for certain types of foods generally characterized by sourness and bitterness, which was probably linked to the different abilities to perceive taste stimuli (e.g., sour and bitter tastes). No differences in food neophobia were highlighted between the two groups. Cohort studies are needed to evaluate the changes in taste abilities and understand their relevance in the progression of the systemic disease.

## Figures and Tables

**Table 1:** Mean taste scores and robust 95% Confidence Intervals (CI) in type 1 diabetes (T1D_ patients and control subjects.

Taste Measurements	T1D Mean (Robust 95% CI)	Control Subjects Mean (Robust 95% CI)	Mean Difference (Robust 95% CI) *	*p*-Value (LRM)
TTS	11.5 (10.6 to 12.5)	13.4 (12.8 to 13.9)	−1.8 (−2.9 to −0.7)	**0.002**
Bitter taste score	2.9 (2.5 to 3.3)	3.5 (3.1 to 3.8)	−0.6 (−1.1 to −0.01)	**0.038**
Salty taste score	3.1 (2.6 to 3.5)	3.4 (3.1 to 3.6)	−0.3 (−0.8 to 0.2)	0.214
Sweet taste score	3.6 (3.5 to 3.8)	3.5 (3.2 to 3.7)	−0.2 (−0.5 to 0.1)	0.221
Sour taste score	2.1 (1.8 to 2.4)	2.9 (2.6 to 3.2)	−0.7 (−1.2 to −0.3)	**0.001**

Significant *p*-values are shown in bold. * Rounded to 1 decimal place. Abbreviations: TTS – Total Taste Score; CI- Confidence Interval; LRM- Linear Regression Model.

**Table 2:** Mean hedonic ratings, mean differences, and robust 95% confidence intervals in T1D patients and control subjects for food categories [42].

Food Categories	T1D Mean (Robust 95% CI)	Control Subjects Mean (Robust 95% CI)	Mean Difference (95% Robust CI) *	*p*-Value (LRM)
Vegetables	4.8 (4.4 to 5.1)	4.1 (3.6 to 4.6)	0.7 (0.1 to 1.3)	**0.023**
Fruits	5.9 (5.5 to 6.2)	5.3 (4.9 to 5.7)	0.6 (0.1 to 1.1)	**0.028**
Starches	5.9 (5.6 to 6.2)	6.0 (5.7 to 6.3)	−0.1 (−0.5 to 0.3)	0.760
Fats and oils	5.0 (4.5 to 5.4)	4.6 (4.1 to 5.1)	0.4 (−0.3 to 1.1)	0.252
Meat and fish	5.5 (5.1 to 5.8)	5.6 (5.3 to 5.9)	−0.1 (−0.5 to 0.4)	0.692
Dairy products	5.5 (5.2 to 5.9)	5.1 (4.7 to 5.5)	0.5 (−0.1 to 1.0)	0.113
Snacks	5.6 (5.2 to 6.0)	5.6 (5.3 to 6.0)	−0.01 ** (−0.6 to 0.5)	0.869

Significant *p*-values are shown in bold. Abbreviations: CI—confidential interval; *n*—number; T1D—type 1 diabetes; LRM—linear regression with robust confidence intervals. * rounded to 1 decimal place; ** rounded to 2 decimal places.

## Supplementary Materials

The following are available online at https://www.mdpi.com/2072-6643/11/12/3052/s1, Table S1: Measurements of T1DM patients. Continuous variables are reported as 50th (median), 25th, and 75th percentiles. Table S2: Measurements of T1D patients and the control group. Continuous variables are reported as 50th (median), 25th, and 75th percentiles.
