# Supplementary material for: Associations Among Taste Perception, Food Neophobia and Preferences in Type 1 Diabetes Children and Adolescents: A Cross-Sectional Study
Source: Nutrients. 2019 Dec 13;11(12):3052. doi: 10.3390/nu11123052 (PMC6950790; doi:10.3390/nu11123052)
Supplement: Supplementary file 1 [file nutrients-11-03052-s001.pdf]

Table S1. Measurements of T1DM patients. Continuous variables are reported as 50th (median), 25th and 75th percentiles. Discrete variables are reported as the number and proportion of subjects with the characteristic of interest.

Measurements	T1D patients (*n* = 31)	Controls (*n* = 31)
**Anthropometric measurements**		
Weight (kg)	41 (37; 53)	33 (29; 44.0)
Height (kg)	1.47 (1.37; 1.64)	1.40 (1.31; 1.52)
BMI (kg/m^2)	19.4 (16.8; 21.6)	17.5 (16.0; 18.4)
Weight (SDS) [27]	0.24 (-0.25; 0.66)	-0.39 (-0.79; 0.29)
Height (SDS) [27]	0.50 (-0.14; 1.12)	0.07 (-0.64; 0.90)
BMI (SDS) [27]	0.13 (-0.54; 0.57)	-0.29 (-1.17; 0.10)
**Tanner stage**		
Prepubertal/early pubertal	14 (45%)	15 (49%)
Pubertal	5 (16%)	6 (19%)
Postpubertal	12 (39%)	10 (32%)
**Therapy**		
MDI therapy	16 (52%)	-
CSII therapy	15 (48%)	-
Duration of diabetes (years)	4 (2,7)	-
$HbA1_c$ (%)*	8 (7; 8)	-
$HbA1_c$ (mmol/mol)	64 (53; 64)	-
Total Cholesterol (mg/dL)	161 (137; 178)	-
HDL-C (mg/dL)	57 (45; 64)	-
LDL-C (mg/dL)	91 (73; 100)	-
Triglycerides (mg/dL)	55 (48; 64)	-

Abbreviations: BMI = Body Mass Index; CSII= continuous subcutaneous insulin infusion; HbA1c = glycated hemoglobin; HDL = high density lipoprotein; LDL = low density lipoprotein; M= male; MDI= multiple daily injections; n= number; SDS = standard deviation score.

Table S2. Measurements of T1D patients and control group. Continuous variables are reported as 50th (median), 25th and 75th percentiles. Discrete variables are reported as the number and proportion of subjects with the characteristic of interest.

Measurements	T1D patients (*n* = 31)	Controls (*n* = 31)
**Taste recognition ability assessment**		
Sweet taste score	4 (3; 4)	4 (3; 4)
Sour taste score	2 (2; 3)	3 (3; 3)
Salty taste score	4 (2; 4)	4 (3; 4)
Bitter taste score	3 (3; 4)	4 (3; 4)
Total taste score (TTS)	12 (10; 13)	14 (12; 14)
PROP responsiveness	18 (0; 54)	38 (6; 95)
**PROP taster status**		
Non-taster	15 (48%)	10 (32%)
Medium-taster	13 (42%)	11 (36%)
Super-taster	3 (10%)	10 (32%)
Fungiform papillae (n/cm2)	13 (9; 19)	24 (19; 28)
Neophobia	20 (15; 25)	20 (15; 27)
**Food preferences assessment**		
Vegetables	5 (4; 5)	4 (3; 5)
Fruits	6 (5; 7)	6 (5; 6)
Starches	6 (5; 7)	6 (5; 7)

Meat and fish	6 (5; 6)	6 (5; 6)
Dairy products	6 (5; 6)	5 (4; 6)
Fats and oils	5 (4; 6)	5 (4; 5)
Snacks	6 (5; 6)	6 (5; 6)
